# Decoding the spatiotemporal heterogeneity of bacterial virulence gene expression using single-cell approaches

**DOI:** 10.3389/fmicb.2026.1832970

**Published:** 2026-06-16

**Authors:** Ângela Alves, Rita Pombinho, Didier Cabanes

**Affiliations:** 1 Instituto de Investigação e Inovação em Saúde - i3S, Universidade do Porto, Porto, Portugal; 2 Group of Molecular Microbiology, Instituto de Biologia Molecular e Celular - IBMC, Porto, Portugal; 3 McBiology Doctoral Program, ICBAS – Instituto de Ciências Biomédicas Abel Salazar, Universidade do Porto, Porto, Portugal

**Keywords:** bacterial pathogenesis, competitive advantage, phenotypic heterogeneity, single-cell analysis, spatiotemporal gene expression, virulence factors

## Abstract

Bacterial pathogenicity arises from dynamic interactions between microbial virulence determinants and host conditions, in which infection outcomes are shaped by both host immunity and phenotypic heterogeneity within clonal bacterial populations. Rather than behaving as uniform entities, bacterial populations diversify into distinct physiological states across space and time during infection. This heterogeneity stems from stochastic gene expression, environmental fluctuations, genetic variation, and cellular aging, generating subpopulations with distinct physiological states. Mechanisms such as bistability, phase variation, persistence, quorum sensing, and history-dependent behavior enable bacteria to diversify phenotypes across space and time, promoting survival under fluctuating and hostile conditions. These strategies underpin cooperative behaviors including division of labor and bet-hedging, which enhance population fitness, virulence potential and resilience during infection. Pathogens exploit heterogeneity to balance acute virulence with long-term persistence, evade host immunity, establish biofilms, and tolerate antibiotic treatment. Recent advances in single-cell technologies, including bacterial single-cell transcriptomics, fluorescent reporter systems integrated with microfluidics, and stable isotope probing, now enable direct measurement of bacterial heterogeneity at unprecedented resolution and within structured environments. Here, we review the molecular mechanisms generating bacterial heterogeneity, the regulatory architectures underlying these processes, and the single-cell technologies that enable their study. Understanding how rare but critical bacterial subpopulations drive infection dynamics will be essential for developing anti-virulence and precision antimicrobial strategies targeting pathogenic subpopulations rather than average population behavior.

## 1 Introduction

Bacterial pathogenicity results from complex interactions between pathogen-associated factors and host conditions. The infection outcome depends not only on the virulence potential of the pathogen but also on the physiological and immunological state of the host. Critical determinants of pathogenic potential include bacterial growth capacity, bacterial localization within the host, tissue tropism, and the ability of bacteria to evade or counteract host defense mechanisms. Virulence factors are typically strain-specific macromolecules involved in adhesion, invasion, proliferation, survival and persistence within the host (Lebrun et al., 2009). However, virulence gene expression is rarely uniform across bacterial populations during infection. Pathogens’ adaptability, which is pivotal to the infection outcome, is achieved by the expression of bacterial virulence factors *per se*, and by the mechanisms employed for their tight and coordinated regulation (Davis et al., 2015; Sturm et al., 2011).

Increasing evidence indicates that bacterial populations behave as heterogeneous ensembles in which individual cells occupy distinct transcriptional and physiological states. Population heterogeneity in bacteria arises from the interplay of multiple factors, including gene expression stochasticity, environmental fluctuations, genetic variation and cellular aging. Stochasticity in gene expression, commonly referred as gene-expression noise, arises from inherent variability in transcription and translation processes, including differences in the mRNA levels, protein stability and regulatory network activity. These variations can lead to phenotypic differences, even among genetically identical bacteria within identical environments. Such variability can produce competitive subpopulations capable of anticipating environmental changes or create non-adaptive states that are naturally eliminated by selective pressure (Cai et al., 2006; Elowitz et al., 2002). Bacterial populations are also shaped by environmental oscillations, including changes in nutrient availability, oxygen gradients, host immune pressure, or exposure to antimicrobials, which selectively favor subpopulations adapted to specific microenvironments (Davis et al., 2015). Genetic variation, including mutations, horizontal gene transfer, and phase variation, also contributes to phenotypic diversification (Haudiquet et al., 2022; Low and van der Woude, 2013). In addition, cellular aging and asymmetric division can generate daughter cells with distinct physiological states, creating non-genetic memory that influences future stress responses and contribute to the emergence of antibiotic-tolerant persister subpopulations (Hossain et al., 2023). Overall, these factors interact to ensure that microbial populations maintain a balance between stability and diversity, thereby optimizing fitness in unpredictable environments and enhancing their ability to adapt, persist and evade host defenses.

Importantly, heterogeneity is inherently spatiotemporal. Spatial structure within host tissues (e.g., oxygen, pH, immune effectors, and nutrient gradients) creates micro-niches that select for distinct bacterial states, while temporal fluctuations during infection drive dynamic state transitions and phenotypic switching. Traditional bulk approaches average these behaviors and therefore obscure rare but clinically relevant subpopulations.

In this review, we examine the mechanistic basis and functional consequences of spatiotemporal heterogeneity in bacterial virulence gene expression. We first discuss the molecular circuits that generate phenotypic diversification, then analyze how heterogeneity enhances virulence through division of labor and bet-hedging strategies. Finally, we critically evaluate emerging single-cell and spatial technologies that are transforming our ability to resolve bacterial behavior in complex host environments.

## 2 Mechanistic basis of spatiotemporal heterogeneity

### 2.1 Stochastic and programmed gene expression

**FIGURE 1:**
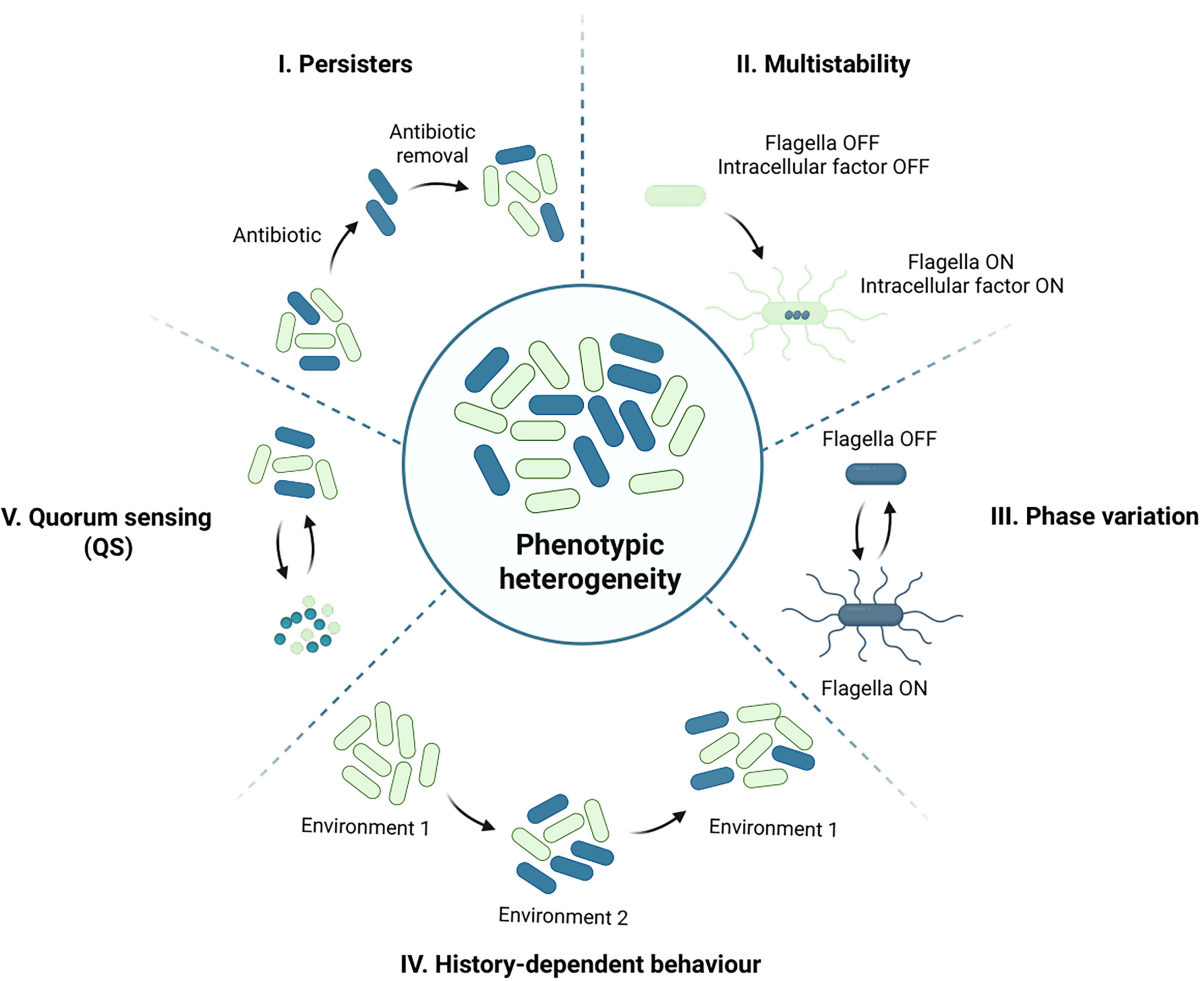
Factors modulating phenotypic heterogeneity in bacterial populations. Clonal bacterial populations can exhibit phenotypic heterogeneity as a result of both intrinsic stochastic processes and extrinsic environmental signals. Intrinsic factors include stochastic gene expression, cell cycle stage, and cellular age, which generate variability among genetically identical cells. Extrinsic signals such as oxygen availability, pH, temperature, nutrient limitation, metabolites, autoinducers, antibiotic stress, and cell–cell interactions further modulate phenotypic diversity. The resulting heterogeneity can lead to distinct functional outcomes within the population. These outcomes are commonly conceptualized as bet-hedging, where phenotypic diversity ensures survival under fluctuating environmental conditions, or division of labor, where specialized subpopulations perform complementary functions that enhance collective fitness and adaptability.

**FIGURE 2:**
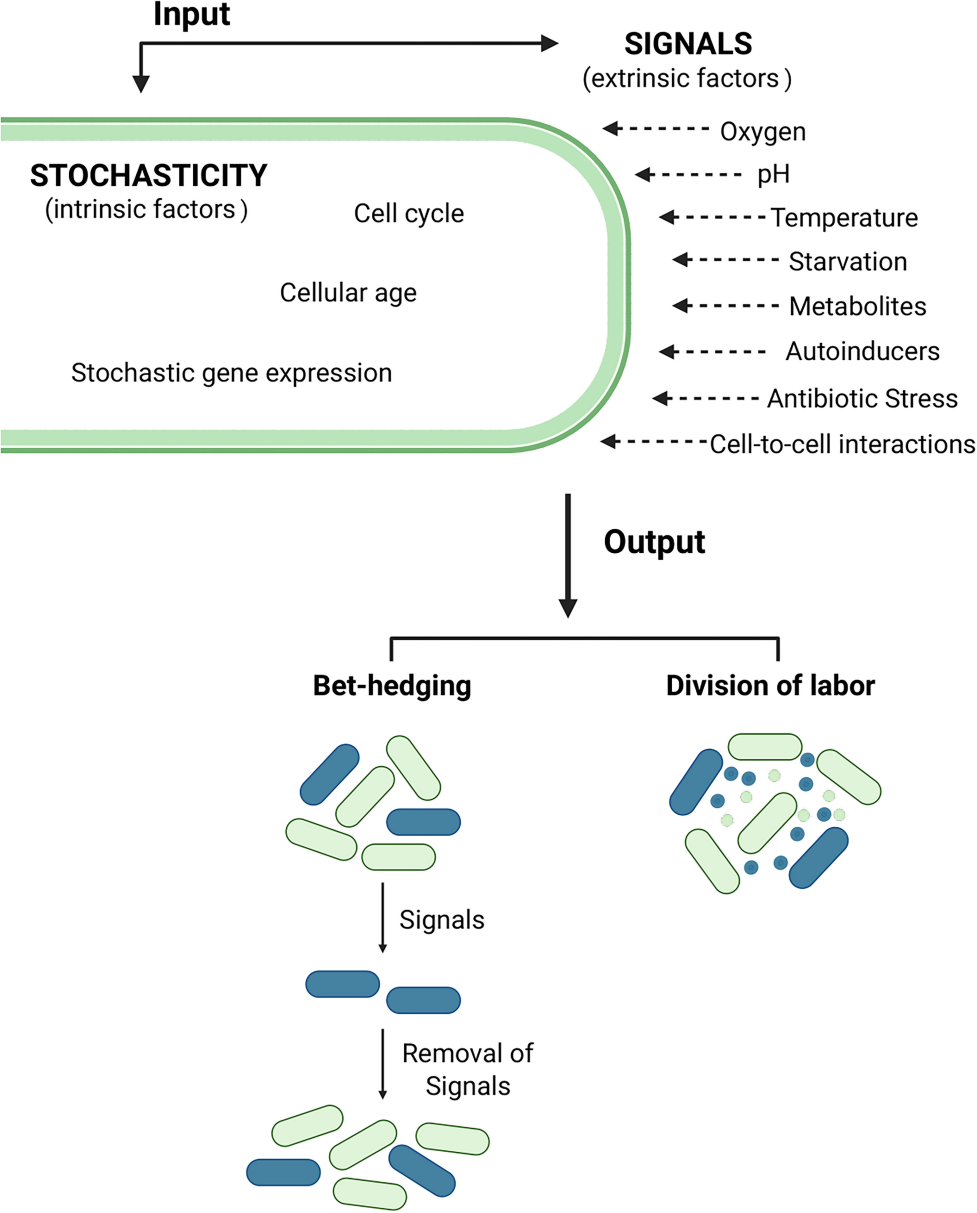
Mechanisms generating phenotypic heterogeneity in bacterial populations. Several regulatory mechanisms contribute to phenotypic heterogeneity, enabling bacterial populations to adapt to environmental challenges and improve survival. I. Persisters: A small subpopulation of cells enters a transient antibiotic-tolerant state. These persister cells survive antibiotic treatment and can resume growth once the antibiotic is removed, thereby repopulating the environment. II. Multistability: Gene regulatory networks can support multiple stable expression states within a genetically identical population. For example, subpopulations may exhibit the Type III secretion system (T3SS) in either an ON or OFF state, allowing flexible responses to environmental changes. III. Phase variation: Reversible switching between phenotypic states (e.g., Flagella ON/OFF) generates diversity within the population and can facilitate adaptation or evasion of host defenses. IV. History-dependent behaviour: Previous environmental exposures influence subsequent cellular responses. When bacteria encounter a previously experienced environment, certain subpopulations respond more rapidly due to their prior physiological state. V. Quorum sensing (QS): As cell density increases, the concentration of autoinducer molecules accumulates. Once a threshold concentration is reached, these signaling molecules bind to bacterial receptors and trigger coordinated changes in gene expression across the population. Blue rectangles represent cells in the OFF state, green rectangles represent cells in the ON state, brown lines indicate the Type III secretion system (T3SS), and blue spheres represent autoinducer molecules. Figures were created using BioRender.

Phenotypic heterogeneity is the phenomenon in which genetically identical bacterial cells display different phenotypes even under homogeneous conditions. Within clonal populations, heterogeneity can arise from intrinsic factors (gene expression noise, cell cycle stage, and cellular age) or from extrinsic factors (microenvironment signals, cell-to-cell interactions either mediated by signaling molecules or physical contact) (Figure 1; Choudhary et al., 2023; Fredriksson and Nyström, 2006; Silander et al., 2012; Špacapan et al., 2020; Wakamoto et al., 2013; Kümmerli et al., 2023). In some cases, heterogeneity arises purely from stochastic fluctuations in gene expression. In other situations, it is generated by regulated genetic circuits capable of producing multiple stable phenotypic states, a phenomenon known as multistability (Figure 2; Gardner et al., 2000; Lopes et al., 2024) The most common form of multistability is bistability, in which cells adopt either high or low expression of specific genes without intermediate states (ON and OFF cells) (Dubnau and Losick, 2006; Laurent et al., 2005). Bistable systems often exhibit hysteresis, meaning that transitions between states depend not only on current conditions but also on previous cellular history. As a result, once virulence or persistence pathways are activated, they may remain active despite minor environmental changes. Such stability is typically generated by regulatory circuits containing positive feedback loops or double-negative regulatory motifs, which amplify small stochastic fluctuations in gene expression (Estell and Schuster, 2025; Ferrell, 2002; Gallie et al., 2015). When transitions between bistable states occur through reversible genetic rearrangements, the phenomenon is known as phase variation. Phase variation controls the expression of numerous bacterial surface structures, including adhesins, capsules, pili, flagella, and iron-acquisition proteins, through mechanisms such as DNA inversions, slipped-strand mispairing, recombination, or epigenetic modifications (Figure 2; Abraham et al., 1985; Anjuwon-Foster and Tamayo, 2017; Bayliss et al., 2001; Levinson and Gutman, 1987; Ren et al., 1999; Seale et al., 2006; Simon et al., 1980; Van Belkum et al., 1999; van Belkum et al., 1998). This illustrates how regulatory architectures convert stochastic fluctuations into stable phenotypic decisions. Importantly, bistable circuits often interact with environmental sensing pathways, quorum-sensing systems, and metabolic feedback loops, generating complex regulatory landscapes that structure bacterial population heterogeneity during infection.

### 2.2 Persistence

Another major driver of phenotypic heterogeneity with direct clinical relevance is bacterial persistence. It is a non-inherited phenomenon that involves the formation of bacterial subpopulations which adopt a slow growth rate and a decreased metabolic activity, being often transiently antibiotic-tolerant (Figure 2; Balaban et al., 2004; Cohen et al., 2013; Levin and Rozen, 2006; Lewis, 2006). Persister cells are one of the major causes of chronic and recurrent bacterial infections, and act as a reservoir for the emergence of antibiotic-resistant strains, posing significant challenges for an effective treatment.

Persistence was also shown to be strongly shaped by host environments. Internalization of *Salmonella enterica* by macrophages induces the rapid formation of a non-replicating persister subpopulation in response to intracellular stresses such as vacuolar acidification and nutrient limitation, leading to phenotypic diversification into replicating and non-replicating cells (Helaine et al., 2014). These macrophage-induced persisters are not merely dormant but can retain metabolic activity and contribute to infection relapse by resuming growth after stress release.

Persistence can be triggered by stressful conditions, including nutrient limitation, intracellular replication, immune system activation and antibiotic exposure (Arnoldini et al., 2014; Liu et al., 2016; Manina et al., 2015). Several molecular mechanisms have been implicated in persister cell formation, including the toxin–antitoxin (TA) system, the stringent response and guanosine tetraphosphate and pentaphosphate ((p)ppGpp) signaling, the SOS response, ATP depletion, among others (Trastoy et al., 2018). TA systems typically consist of a stable toxin (a protein) that disrupts essential physiological processes (e.g., DNA replication, transcription, cell wall synthesis, cell division), and a labile anti-toxin (either RNA or a protein) that binds and neutralizes the toxin. Under normal conditions, the antitoxin is expressed continuously and binds the toxin, avoiding cell damage. However, under stress conditions, the antitoxin is rapidly degraded, leading the toxin free to inhibit critical cellular processes and therefore induce a state of bacterial dormancy against which antibiotics are ineffective (Lewis, 2010; Maisonneuve et al., 2013; Moyed and Bertrand, 1983; Ramisetty et al., 2016; Schuster and Bertram, 2013; Van Melderen and Wood, 2017; Wang and Wood, 2011). There are eight groups of TA systems, depending on the molecular nature of the antitoxin and the mechanism of toxin neutralization, being persistence mostly associated with type I and II (Harms et al., 2018). Importantly, persistence reflects antibiotic tolerance rather than genetic resistance, although persister reservoirs can facilitate the subsequent evolution of resistance.

The stringent response is mediated by the stress alarmone (p)ppGpp, which is produced by RelA or SpoT in response to nutrient starvation, pH changes and heat shock. Acting as a global signaling molecule, (p)ppGpp reprograms transcription and modulates protein activity, thereby promoting slow growth or dormancy under environmental stress (Irving et al., 2020; Potrykus and Cashel, 2008; Srivatsan and Wang, 2008). In *Escherichia coli* (*E. coli*), (p)ppGpp inhibits DNA primase, proteins involved in lipid metabolism and nucleotide metabolism (Gaca et al., 2015; Kanjee et al., 2012) and rRNA synthesis (Shimada et al., 2013). Interestingly, (p)ppGpp also regulates TA systems. The ten mRNase-encoding TA systems in *E. coli* were found to be upregulated in response to amino acid starvation, a signal that induces the production of (p)ppGpp (Maisonneuve and Gerdes, 2014; Shan et al., 2017). Mutants lacking relA or relA and spoT form fewer persisters due to reduced levels of (p)ppGpp (van den Bergh et al., 2017).

An example is the HipBA TA system in *E. coli* (Black et al., 1994; Moyed and Bertrand, 1983). The HipA toxin functions as a serine/threonine kinase that inhibits cell growth, while the HipB antitoxin neutralizes HipA activity. *E. coli* HipA inactivates the glutamyl-tRNA synthetase GltX, leading to the accumulation of uncharged tRNAs sensed by the ribosome-associated protein RelA, which synthesizes the alarmone (p)ppGpp, and signals amino acid starvation. (p)ppGpp binds to target proteins, such as RNA polymerase and primase, reprograms transcription, downregulates macromolecular synthesis, and ultimately induces dormancy (Amato and Brynildsen, 2015; Bokinsky et al., 2013; Germain et al., 2013; Hauryliuk et al., 2015; Korch et al., 2003; Semanjski et al., 2018).

DNA damage triggers the SOS response, a major survival pathway controlled by two regulatory proteins, the repressor LexA and the inducer RecA. Under normal conditions, LexA binds within the promoter regions of SOS genes, repressing them. However, when DNA damage occurs, RecA binds to the accumulated ssDNA and becomes activated, thereby inducing the autocleavage and inactivation of LexA, and leading to the derepression of SOS genes (Kovačič et al., 2013). The SOS response does not activate the population homogeneously, thus actively contributing to the generation of subpopulations with different stress tolerances and mutagenic capacities. Some antibiotics can cause DNA damage that triggers SOS pathway, which in turn promotes persister cell formation through TA modules (Maslowska et al., 2019). TA systems and the general stress response were considered the primary drivers of persister formation, but this view has been challenged. In *S. aureus*, mutants lacking key tricarboxylic acid (TCA) cycle enzymes lead to increases in persister formation accompanied by lower ATP levels (Zalis et al., 2019), while deletion of TA modules or (p)ppGpp synthase had no significant effect on persistence (Conlon et al., 2016). In *E. coli*, chemical depletion of ATP using arsenate or cyanide-chlorophenylhydrazone increased the proportion of persisters (Kwan et al., 2013; Shan et al., 2017). It is speculated that low intracellular ATP levels downregulate cellular physiological processes such as transcription and translation, leading to inactivation of antibiotic targets and multidrug tolerance.

Further studies revealed that persister formation in *Salmonella* is linked to TA systems, including acetyltransferase toxins that inhibit translation through tRNA acetylation, thereby promoting growth arrest during macrophage infection (Cheverton et al., 2016; Rycroft et al., 2018). Notably, macrophage internalization can increase persister frequency by several orders of magnitude across clinical isolates, indicating that host-induced persistence is a conserved feature of pathogenic *Salmonella* strains.

### 2.3 Quorum sensing (QS)

Quorum sensing is a density-dependent gene regulation mechanism in bacteria, mediated by small signaling molecules known as autoinducers. This communication system allows bacteria to coordinate collective behaviors in response to changes in population density (Figure 2; Miller et al., 2002). As the bacterial population grows, the autoinducer concentration rises. Once it surpasses a critical threshold, the autoinducers bind to specific receptors, triggering changes in gene expression that modulate collective population behaviors. The chemical nature of autoinducers depends on the producing organism. For instance, Gram-negative bacteria produce small molecules that interact with cytoplasmic transcription factors or transmembrane two-component histidine sensor kinases, whereas Gram-positive bacteria typically produce oligopeptides and rely on transmembrane two-component histidine sensor kinases as receptors (Novick and Geisinger, 2008; Papenfort and Bassler, 2016). The autoinducer-receptor complex often activates the expression of the gene encoding autoinducer synthase, thus establishing a positive feedback loop that amplifies signal production. QS regulation controls several bacterial traits, including bioluminescence (Anetzberger et al., 2009; Bassler et al., 1994; Engebrecht et al., 1983; Xavier and Bassler, 2003), biofilm formation (Hammer and Bassler, 2003; Miller et al., 2002), natural competence for DNA uptake (Kleerebezem et al., 1997; Okada et al., 2005), secondary metabolite biosynthesis (Barnard et al., 2007), and virulence (Bronesky et al., 2016; Da Silva and De Martinis, 2012; De Kievit and Iglewski, 2000; Garmyn et al., 2011; Gray et al., 2006; Miller et al., 2002; Nuss et al., 2016; Personnic et al., 2018). QS can also drive phenotypic heterogeneity within bacterial populations. Some cells may enter an active QS state (ON), while others remain in an inactive state (OFF), leading to the formation of different microbial subsets (Anetzberger et al., 2009; Bauer et al., 2017; Cárcamo-Oyarce et al., 2015; Plener et al., 2015). This heterogeneity can be further shaped by the presence of “cheaters,” bacteria that benefit from QS-regulated public goods while avoiding the metabolic cost associated with their production (Allen et al., 2016). Recent single-cell studies demonstrate that such heterogeneity in QS and public-goods expression is not purely stochastic but is shaped by social interactions, metabolic state, and spatial organization within bacterial populations. In *Pseudomonas aeruginosa*, significant cell-to-cell variability and bimodality in siderophore gene expression have been observed, with coordination emerging over time through regulatory feedback and signaling (Mridha et al., 2022). Moreover, spatial positioning and lineage relationships contribute to structured differences in gene expression between individual cells, indicating that phenotypic heterogeneity is influenced by both intrinsic regulatory mechanisms and population-level interactions (Mridha et al., 2024). These findings highlight that heterogeneity in gene expression can reflect collective decision-making processes within bacterial communities rather than solely random fluctuations. *Staphylococcus aureus* (*S. aureus*) is an opportunistic pathogen that causes bacteremia and endocarditis (Kwiecinski and Horswill, 2020). During infection, *S. aureus* splits into two distinct subpopulations: one promotes biofilm formation contributing to chronic infections, while the other remains planktonic and secretes toxins causing acute infection (Boles and Horswill, 2008). This bimodal switch is regulated by the agr quorum-sensing system, which is activated in response to the self-produced extracellular signal AIP (autoinducing peptide) (Ji et al., 1997). When extracellular AIP concentration reaches a critical threshold, it binds to the membrane-bound histidine kinase receptor AgrC and activates its cognate regulator AgrA via phosphorylation. AgrA∼P upregulates hemolytic toxin-encoding genes (hla, hlb, hlg) and phenol-soluble modulins (psmα and psmβ) responsible for bacterial dispersion and acute bacteremia (Peschel and Otto, 2013; Recsei et al., 1986; Saïd-Salim et al., 2003; Thoendel et al., 2011). In contrast, AgrA∼P downregulates the icaADBC operon, required for the synthesis of the extracellular polysaccharide matrix, as well as adhesion proteins such as SpA, that mediate cell aggregation and attachment during biofilm formation (Boles and Horswill, 2008; Peng et al., 1988; Recsei et al., 1986). Spatial structure, particularly in biofilms, further amplifies QS heterogeneity by limiting signal diffusion and creating localized activation thresholds. Biofilms protect bacteria from antibiotic therapy and host immune defenses, contributing to bacterial persistence and chronic infections.

### 2.4 History-dependent behavior (HDB)

History-dependent behavior (HDB) or memory refers to the phenomenon in which bacteria respond to current environmental conditions based on prior experiences. It enables rapid adaptation and survival to environmental fluctuations (Figure 2; Zhang et al., 2023). HDB should be understood as a broad functional concept describing how past cellular states influence present phenotypes, encompassing both transient and heritable mechanisms. Short-term memory encompasses transient and not inheritable mechanisms, including transcriptional memory, in which bacterial cells previously exposed to a stimulus exhibit faster recruitment of transcription factors, enabling rapid adaptation to environmental fluctuations (Zhang et al., 2023). Long-term memory can arise through multiple mechanisms, including protein-based inheritance, where stress-induced protein aggregates or stable molecular states are asymmetrically passed to daughter cells, persisting across generations and providing survival advantages like high reproductive rates, fast recovery and enhanced tolerance (Wolf et al., 2008; Zhang et al., 2023). Epigenetic regulation, such as DNA methylation, represents a specific mechanistic subset of HDB, enabling bacteria to transmit information about the phenotypic regulatory state of the parent cell to offspring and acting as a hidden driver of persister formation (Adam et al., 2008). Thus, while epigenetic mechanisms encode heritable regulatory states, HDB also includes non-epigenetic processes such as regulatory circuit hysteresis, metabolic states, and protein inheritance, which collectively contribute to phenotypic heterogeneity. Such memory effects are increasingly recognized as important contributors to infection dynamics, enabling rapid reactivation of virulence programs upon re-encounter with host-like conditions.

In *E. coli*, a heritable iron memory can connect diverse stress responses, including swarming, biofilm formation and antibiotic survival. Switching between iron memory states may therefore maximize survival in varied environments. Low intracellular iron levels act as a signal for swarming and are positively correlated with increased survival to antibiotics. In contrast, high iron is a signal for biofilm formation. Antibiotics trigger the production of ROS, and high iron levels further increase ROS, increasing lethality. A multigenerational iron memory may enhance the survival chances of at least some individuals within the population under antibiotic stress (Bhattacharyya et al., 2023).

## 3 Benefits of microbial heterogeneity for virulence

Phenotypic heterogeneity generates emergent properties at the population level that cannot be predicted from the behavior of individual cells. Interactions among specialized subpopulations enable cooperative behaviors that enhance survival, virulence, and resilience to environmental stress. One emergent property is population-level robustness. By maintaining multiple phenotypic states simultaneously, bacterial populations can rapidly adapt to fluctuating environments. Another emergent property is collective functionality through division of labor, where specialized subpopulations perform complementary roles such as toxin production, extracellular matrix synthesis, or metabolic adaptation (Ackermann, 2015; Kolter and Greenberg, 2006). Bacteria are capable to produce public goods made by some cells that are secreted into the environment to be easily accessed by non-producers (cheaters). Public goods involve collective benefits but require tight mechanisms to avoid community collapse by cheaters, while private goods ensure individual benefits, maintaining the baseline fitness (Menon and Korolev, 2015). Phenotypic diversity can benefit cell populations by enabling cooperative behaviors (such as division of labor or goods sharing) and by producing specialized cells that gain fitness advantages under challenging conditions (bet-hedging) (Figure 1). These cooperative interactions allow microbial communities to optimize resource allocation while maintaining adaptability during infection. Consequently, bacterial virulence should be viewed not only as a property of individual cells but also as an emergent behavior of heterogeneous populations.

### 3.1 Division of labor

Bacteria have evolved a smart strategy, known as division of labor, in which distinct subpopulations within a microbial community specialize in carrying out different tasks that are essential for the overall population survival (West and Cooper, 2016). This social cooperative behavior may occur in one direction, when individuals of one phenotype altruistically help other phenotype, or in both directions, when individuals of each phenotype help each other for their mutual benefit (West and Cooper, 2016). This bacterial adaptive strategy does not require a change in the environment (Zhang et al., 2016).

*Bacillus subtilis* is a spore-forming Gram-positive bacterium. Its cell differentiation is triggered by environmental signals that trigger the phosphorylation of the master regulator Spo0A through sensor histidine kinases. Spo0A is essential for motility, sporulation and matrix production during biofilm formation (Hamon and Lazazzera, 2001; Lopez et al., 2009; Vlamakis et al., 2013). The levels of activated regulator, Spo0A∼P, demonstrate a broad heterogeneity among individual cells within the population (Chastanet et al., 2010). In bacteria with low levels of Spo0A∼P, cell motility is promoted (Verhamme et al., 2007), while in bacteria with intermediate levels of Spo0A∼P, the production of two matrix components (EPS and TasA) is induced leading to biofilm production (Chai et al., 2011; Fujita et al., 2005). In turn, in bacteria with high levels of Spo0A∼P, the production of matrix is repressed and simultaneously genes involved in sporulation are activated (Vlamakis et al., 2008). Cell differentiation in *B. subtillis* is perceived as an adaptive strategy that enables the population to either proliferate or persist under changing environmental conditions. A highly differentiated heterogeneous population allows expression of a distinct set of genes in different cell types.

*Pseudomonas aeruginosa* is an opportunistic human pathogen that causes chronic infections due to its ability to form biofilms, where the bacteria are present in aggregates encased in a self-produced extracellular matrix (Ciofu and Tolker-Nielsen, 2019). This matrix can be composed of extracellular DNA (eDNA), proteins (e.g., adhesin CdrA, which functions as a matrix-stabilizing protein), RNA, exopolysaccharides (e.g., Pel and Psl), water, lipids and/or extracellular membrane vesicles (Chiba et al., 2022; Fleming et al., 2017). The *cdrA*, *pel* and *psl* genes are transcriptionally activated under high levels of c-di-GMP (Borlee et al., 2010; Starkey et al., 2009). *P. aeruginosa* employs a protein signaling complex known as the Wsp system, which is a surface-sensing mechanism involved in the initial attachment of planktonic cells to biotic or abiotic surfaces. This first step of biofilm formation leads to high cellular c-di-GMP levels, thus stimulating the production of adhesins and extracellular matrix components crucial for biofilm formation and repression of flagellar motility genes. The bacterial subpopulation with low c-di-GMP levels potentiates surface motility and engages bacteria in a planktonic state for them to explore a new surface for attachment (Almblad et al., 2019). These two subpopulations - high c-di-GMP polysaccharide producers and low c-di-GMP surface explorers - are both essential for efficient biofilm establishment, representing a division of labor that enhances early biofilm formation (Armbruster et al., 2019).

Infection by *Salmonella* spp., a major enteric pathogen, also exploits phenotypic heterogeneity to escape from host immune detection and response. During infection, *Salmonella enterica* serovar Typhimurium invades non-phagocytic epithelial cells in the small intestine, making use of a type III secretion system (T3SS), which forms a complex that injects bacterial effector proteins into the host cell cytosol. Clonal populations of *Salmonella* within the gut differentiate into two subpopulations, with only one expressing a set of T3SS-1 virulence genes, under an ON or OFF regulatory pattern (Hautefort et al., 2003). The T3SS-1 ON subpopulation invades the intestinal epithelium and causes inflammation (Stecher et al., 2007). In contrast, the T3SS-1 OFF subpopulation remains non-invasive, but benefits from the inflammatory environment generated by the ON bacteria, thereby allowing T3SS-1 OFF cells to outcompete gut microbiota and promote their proliferation and potential transmission to new hosts (Thiennimitr et al., 2011; Winter et al., 2010). Another strategy used by *Salmonella* to overcome host defense mechanisms is based on the heterogeneous expression of the flagellin gene *fliC*. During infection, *Salmonella enterica* serovar Typhimurium expresses *fliC* in lymphoid tissue (Peyer’s patches - PP), but not at systemic sites such as mesenteric lymph nodes and spleen, being this differential expression a pathogenic advantage. Bacteria expressing FliC in the PP (*fliC* ON) stimulate the innate immune system and trigger a strong inflammatory response, whereas bacteria lacking FliC expression (*fliC* OFF) escape host immune detection in the intestine and disseminate to systemic sites of replication (Cummings et al., 2006; Stewart et al., 2011).

*Streptococcus pneumoniae*, a Gram-positive pathogen and the leading cause of bacterial pneumonia in children and the elderly worldwide, also employs a division of labor strategy involving the toxin PezT encoded by the pezAT toxin-antitoxin (TA) system. PezT inhibits cell wall synthesis by catalyzing the phosphorylation of the uridine diphosphate N-acetylglucosamine (UNAG) in an ATP-dependent manner, converting it into UNAG-3’-P (Mutschler et al., 2011). Since UNAG is the universal precursor of the sugar peptidoglycan backbone in bacterial cell wall, the PezT-mediated UNAG-3’-P product acts as a competitive inhibitor of MurA, an enzyme catalyzing the first step of peptidoglycan biosynthesis. Under normal growth conditions, the PezA antitoxin binds and neutralizes PezT. However, under stress conditions such as nutrient limitation, PezA is degraded and active PezT is released, which consequently inhibits cell wall synthesis, leading to cell lysis and toxin release (Chan and Espinosa, 2016). On a bacterial population, metabolically inactive persister cells and slowly dividing cells survive this process, whereas rapidly dividing cells, which require continuous murein synthesis, undergo lysis and release cytosolic pneumolysin, a major virulence factor of *S. pneumoniae*. Moreover, partial cell lysis and inhibition of capsular polysaccharide synthesis by UNAG-3’-P promote biofilm formation. When the stress conditions are relieved, the surviving cells resume PezA production, neutralizing PezT activity and thus reestablishing the PezAT system equilibrium (Chan et al., 2012; Mutschler et al., 2011).

### 3.2 Bet-hedging

Alongside with the division of labor, bet-hedging is one of the main beneficial strategies for population survival. Bet-hedging is a strategy in which phenotypic heterogeneity does not provide an immediate advantage but enhances long-term survival in fluctuating environments. In the bet-hedging strategy, the overall fitness of the population is maximized at the expense of certain individuals, as different subpopulations express distinct traits without sensing environmental changes. As the environment fluctuates, at least one subpopulation will be better adapted and will survive, ensuring the persistence of the lineage (Beaumont et al., 2009; Schreiber et al., 2016).

In *Yersinia pseudotuberculosis*, an enteric pathogen responsible for a variety of clinical symptoms in humans, the bistable expression of the virulence regulator RovA functions as a bet-hedging strategy, allowing bacterial population to preadapt to the dynamic conditions encountered during different stages of infection. The thermo-responsive transcriptional activator RovA regulates the expression of the major epithelial invasion factor and several genes associated with metabolism and stress response at 25 °C. However, at 37 °C, a conformational change in RovA dimerization domain reduces its DNA-binding affinity and increases its proteolytic degradation by the Lon protease, shifting the system toward the OFF state (Bücker et al., 2014; Heroven et al., 2004; Nagel et al., 2001). In the early stages of infection, bacteria express RovA and invasin, promoting their internalization into M-cells. Once inside the Peyer’s patches, most bacteria switch off *rovA* expression to evade host immune defenses. Importantly, a bacterial subpopulation still expresses RovA, allowing *Yersinia* to survive and persist in the intestinal lumen and to potentiate reinfection via invasin. Controlling the ON/OFF ratio of RovA expression in the bacterial population allow to fine-tune virulence determinant expression to optimize adaptation during different infection stages (Herbst et al., 2009; Nuss et al., 2015, 2016).

In *E. coli*, the production of colicins provides a clear example of bet-hedging. Colicins are proteins produced by *E. coli* that kill competing bacteria through several mechanisms, including pore formation in the cytoplasmic membrane, nucleic acid degradation or murein and lipopolysaccharide biosynthesis inhibition (Cascales et al., 2007). Colicinogenic populations are phenotypically heterogeneous, with only a small subpopulation stochastically expressing colicins at a high fitness cost, as toxin release requires cell lysis. The majority of cells remain in a colicin-silenced state. This spontaneous phenotypic switching between colicin-silencing and expressing states allows the population to balance the cost of colicin production with the need to repel its competitors, thereby increasing long-term fitness in fluctuating competitive environments (Bayramoglu et al., 2017).

## 4 Methodological approaches to access bacterial heterogeneity at the single-cell level

Clonal bacterial populations exhibit phenotypic heterogeneity, which facilitates their survival and adaptation to unpredictable environments. Although conventional bulk analysis is valuable for extrapolating the global behavior of bacterial populations, understanding their dynamics requires resolving at the level of individual cells. Single-cell techniques, including single-cell transcriptomics, microscopy, flow cytometry and mass spectrometry imaging, provide spatial and temporal resolution to study bacterial heterogeneity. These techniques, summarized in Table 1, are discussed in the following section.

**TABLE 1:** Comparison of single-cell approaches to study bacterial population heterogeneity.

Techniques	Advantages	Disadvantages	Microorganisms	References
MATQ-seq	Probe-independent approach Low cell drop-out rate	Physical separation and lysis of single bacterium Low sensitivity (150–200 genes) Low cell throughput (hundreds of cells) High costs	*S. enterica* *P. aeruginosa*	Imdahl et al., 2020; Homberger et al., 2023
microSPLIT	Probe-independent approach High cell throughput (thousands of cells) Moderate costs	Involves fixation and permeabilization of bacterium Low sensitivity (138–230 genes) High cell drop-out rate	*B. subtilis* *E. coli*	Kuchina et al., 2021
PETRI-seq	Probe-independent approach High cell throughput (thousands of cells) Moderate costs	Involves fixation and permeabilization of bacterium Low sensitivity (103 operons) High cell drop-out rate	*E. coli* *S. aureus*	Blattman et al., 2020
M3-seq	Probe-independent approach High cell throughput Improved rRNA depletion	Lower transcript rate High costs	*B. subtilis* *E. coli*	Wang et al., 2023
BacDrop	Probe-independent approach High cell throughput	Low mRNA yield Limited applicability in complex bacterial communities	*K. pneumoniae*	Ma et al., 2023
ProBac-seq	High cell throughput (thousands of cells) High sensitivity (2959–4181 genes)	Probe-dependent approach No transcriptome-wide coverage	*B. subtilis* *E. coli* *C. perfringens*	McNulty et al., 2023
par-seqFISH	High temporal and spatial resolution High cell throughput (thousands of cells) Low cell drop-out rate Moderate costs	Probe-dependent approach Fixation and permeabilization step Low sensitivity (105 genes)	*P. Aeruginosa* *E. coli*	Dar et al., 2021
NanoSIMS	High sensitivity to measure the isotopic composition of single cells Spatial resolution	Low cell throughput No subsequent downstream applications (sample is destroyed)	*E. coli* *K. oxytoca*	Nikolic et al., 2017; Schreiber et al., 2016
Raman microspectroscopy	No cultivation required Non-destructive approach High resolution	Low sensitivity and weak signal Low cell throughput High cost	*B. thuringiensis* *E. coli*	Chen et al., 2006; Wang et al., 2022

### 4.1 Bacterial single-cell transcriptomics

Single-cell transcriptomics has revolutionized biology through the analysis of gene expression at the individual cell resolution level. However, its application to bacterial populations remains limited due to several technical challenges: (i) Bacterial cells contain only femtogram amounts of RNA (Milo and Phillips, 2015), which is 100 times less than in eukaryotic cells (Milo and Phillips, 2015); (ii) Bacterial messenger RNA (mRNA) is intrinsically labile, with a short half-life on the scale of minutes, compared with hours in eukaryotes (Rauhut and Klug, 1999); (iii) Accessing mRNA from single cells requires cell lysis, which is difficult in bacteria due to their cell wall; (iv) Bacterial transcripts lack a 3′ poly-Adenosine (poly(A)) tail, precluding the use of poly-T primers in reverse transcription (RT) for selective mRNA enrichment and depletion of ribosomal RNA (rRNA), which composes 98% of the bacterial transcriptome (Giannoukos et al., 2012; Westermann et al., 2016); (v) Whereas most of the current eukaryotic single-cell RNA-sequencing (scRNA-seq) protocols have a transcript detection limit of 10 copies per cell (Bagnoli et al., 2018; Ziegenhain et al., 2017), prokaryotic scRNA-seq implies lower average of mRNA copies (0.4 copies/cell) (Bartholomäus et al., 2016; Taniguchi et al., 2010).

To overcome these technical barriers in bacterial single-cell transcriptomics, some scRNA-seq techniques have been described, including Multiple Annealing and deoxycytidine (dC) Tailing-based Quantitative scRNA-seq (MATQ-seq), combinatorial barcoding methods such as microbial Split-Pool Ligation Transcriptomics (microSPLiT) and Prokaryotic Expression Profiling by Tagging RNA *In situ* and sequencing (PETRI-seq), and Probe-based Bacterial sequencing (ProBac-seq). Massively parallel droplet-based methods, such as M3-seq and BacDrop have also emerged. Besides bacterial scRNA-seq, individual gene expression can also be combined with spatial information using Parallel Sequential Fluorescence *in situ* Hybridization (par-seqFISH) (Blattman et al., 2020; Dar et al., 2021; Homberger et al., 2023; Imdahl et al., 2020; Kuchina et al., 2021; McNulty et al., 2023).

#### 4.1.1 Multiple Annealing and Tailing-based microbial scRNA-seq (MATQ-seq)

Multiple Annealing and deoxycytidine (dC) Tailing-based Quantitative scRNA-seq isolates bacterial cells into microwells using fluorescent-activated cell sorting (FACS), enabling efficient separation of thousands of cells within short time. Following enzymatic lysis of single cells to release total RNA, reversion transcription (RT) is performed using a combination of random hexamer and Multiple Annealing and Looping-based Amplification Cycles (MALBAC) primers. These primers enable high-efficiency hybridizing with transcripts even at low temperatures, allowing sensitive detection of low-abundant transcripts. The RT reaction is followed by dC-tailing, which facilitates efficient second-strand synthesis using primers that anneal to the poly(C) tail and incorporate unique molecular identifiers (UMIs) in the form of random hexamers. Thereafter, the cDNA is amplified and used as a template for library preparation. MATQ-seq provides absolute and unbiased transcript counts. Recent improvements of this procedure include the use of a more efficient reverse transcriptase (SuperScript IV) and an rRNA depletion step that reduces cell loss and improves transcript capture rates and gene coverage (Bartholomäus et al., 2016; Homberger et al., 2023; Imdahl et al., 2020). MATQ-seq was successfully applied to *S. enterica* and *P. aeruginosa* grown under different conditions (NaCl and anaerobic shock). Indeed, scRNA-seq enabled the detection of small regulatory RNAs, such as GcvB or CsrB, at the single-cell level and confirmed the phenotypic heterogeneity within the clonal population regarding the expression of pathogenicity-related genes and flagellar components (Homberger et al., 2023). Overall, the low rate of cell loss and high sensitivity of gene detection make MATQ-seq well-suited for studies with limited input material, such as analysis of small bacterial populations in host niches or intracellular bacteria. However, it is limited to a few hundred bacteria and has relatively high associated costs (Homberger et al., 2023; Imdahl et al., 2020).

Despite its sensitivity, MATQ-seq remains limited by relatively low throughput compared with barcoding-based methods and requires specialized cell sorting and library preparation steps that increase cost and experimental complexity. Additionally, transcript capture efficiency may vary between cells due to differences in lysis efficiency or RNA degradation, potentially introducing technical noise that complicates quantitative comparisons across single cells.

#### 4.1.2 Barcoding-based scRNA-seq of microbes (PETRI-seq and microSPLiT)

Combinatorial barcoding methods (PETRI-seq and microSPLiT) enable the discrimination of thousands of individual cell transcriptomes, eliminating the need of physical single-cell isolation. Both techniques use combinatorial indexing to achieve scRNA-seq of a bacterial population. Through these techniques, bacteria are immobilized by fixation and permeabilized with lysozyme for *E. coli* or lysostaphin for *S. aureus* (PETRI-seq) or with a combination of lysozyme and Tween-20 (microSPLiT). Before RT, transcripts are poly-adenylated or the RT is initiated using random primers (Blattman et al., 2020; Kuchina et al., 2021). cDNA is successively barcoded at each round, giving to each bacterial cell a unique identity. Barcoding is followed by cell lysis, library preparation and sequencing. MicroSPLiT includes an enrichment of mRNAs with polyadenylate polymerase (PAP) so that rRNA is not carried along with the reaction (Blattman et al., 2020; Kuchina et al., 2021). A recent study that employs MicroSPLiT in *E. coli* and *B. subtilis* successfully distinguished a heat-shocked from a non-heat-shocked subpopulation and identified a rare cell state induced by cellular stress, demonstrating its ability to detect differential gene expression among the overall population (Kuchina et al., 2021). A PETRI-seq study examining various bacterial growth conditions uncovered growth-dependent *E. coli* subpopulations, based on their unique single-cell transcription profiles. When applied to *S. aureus*, PETRI-seq identified a rare subpopulation of bacterial cells undergoing prophage induction (Blattman et al., 2020). MicroSPLiT and PETRI-seq analyze thousands of bacterial cells simultaneously, offsetting the lower transcript capture rate and a higher rate of cell loss. While MATQ-seq exhibits lower cell loss, barcode-based combinatorial methods provide much higher throughput, enabling broader population profiling (Homberger et al., 2022).

A major limitation of combinatorial barcoding approaches lies in their relatively low transcript capture efficiency compared with eukaryotic scRNA-seq methods. Because bacterial cells contain extremely small amounts of RNA, many transcripts remain undetected, resulting in sparse datasets that can complicate downstream interpretation and clustering analyses. In addition, fixation and permeabilization steps may introduce biases that affect transcript accessibility.

#### 4.1.3 Droplet-based scRNA-seq (M3-Seq and BacDrop)

Droplet-based scRNA-seq approaches, such as M3-seq and BacDrop, further extend combinatorial barcoding with microfluidic encapsulation, enabling transcriptome-wide profiling of hundreds of thousands of individual bacterial cells. The Massively-parallel Microbial mRNA Sequencing (M3-Seq) method combines a two-step procedure of combinatorial-fluidic indexing (plate-based *in situ* barcoding and droplet-based barcoding) and a post hoc rRNA depletion, allowing high-throughput mRNA profiling across hundreds of thousands of single bacterial cells. M3-seq revealed independent phage induction programs in *B. subtilis* and an acid-tolerant *E. coli* subpopulation (Wang et al., 2023). In parallel, another droplet-based genome-wide massively parallel bacterial scRNA-seq technology, BacDrop, was reported. BacDrop was applied to study *Klebsiella pneumoniae* clinical isolates and revealed transcriptionally distinct subpopulations associated with different outcomes, including antibiotic persistence (Ma et al., 2023).

BacDrop performs rRNA depletion *in situ*, while M3-seq performs rRNA depletion after library amplification, reducing the risk of losing unamplified and non-rRNA transcripts. Both M3-seq and BacDrop provide a high-throughput analysis but are constrained by high sequencing costs and their limited applicability in studying complex bacterial communities, such as microbiota samples (Ma et al., 2023; Wang et al., 2023). Probe-based scRNA-seq methods offer a complementary strategy, combining targeted DNA probes with droplet microfluidics.

Although droplet-based approaches dramatically increase throughput, they also involve substantial sequencing costs and complex library preparation workflows. Furthermore, droplet encapsulation efficiency and uneven transcript capture may lead to gene dropout events, particularly for low-abundance transcripts. These challenges currently limit their routine application to large-scale or highly complex microbial communities.

#### 4.1.4 Probe-based scRNA-seq (ProBac-seq)

Recently, a probe-based bacterial sequencing (ProBac-seq) method was described to conduct bacterial single-cell RNA sequencing. This method combines *in situ* hybridization of DNA probes with 10× Genomics’ Chromium Controller, a commercial high-throughput droplet-based microfluidic device that resolves the mRNA profile of thousands of bacterial cells. Using this approach, distinct cellular states of *B. subtilis* were successfully identified, including genetic competence and sporulation, as well as a subpopulation of *E. coli* producing fimbriae. In *C. perfringens* population, a heterogeneity was also revealed with regard to toxin secretion in response to acetate (McNulty et al., 2023). ProBac-seq provides high-throughput, high-resolution and low-cost, scRNA-sequencing, being a complement to other scRNA-seq methods. However, as it relies on a microfluidic-based approach, it may encounter “gene dropout,” especially for genes with lower expression levels, which may lead to an incomplete and biased transcriptome profile (Samanta et al., 2024; Wang et al., 2021).

Because ProBac-seq relies on targeted hybridization probes, it requires prior knowledge of the genome and limits the analysis to predefined transcripts. Consequently, this approach is less suitable for unbiased transcriptome discovery or for studying poorly characterized organisms. Probe design and hybridization efficiency can also influence transcript detection sensitivity.

#### 4.1.5 Single-cell spatial transcriptomics using combinatorial fluorescence (Par-seqFISH)

Besides bacterial scRNA-seq, gene expression analysis can also be combined with spatial transcriptomic information using Parallel Sequential Fluorescence *in Situ* Hybridization (par-seqFISH). First, specific mRNA sequences are hybridized by primary non-fluorescent probes flanked by short gene-specific sequences. Then, transcripts can be detected via secondary hybridization with a fluorescent-labeled readout probe targeting the first probe. By employing different sets of readout probes labeled with distinct fluorophores and several rounds of hybridization, several genes can be visualized simultaneously in a single sample. The sequentially acquired data can generate multigene profiles at the single-cell level with spatial information (Dar et al., 2021). Par-seqFISH was applied to investigate the mRNA expression profiles of approximately 10^5^ single *P. aeruginosa* cells grown under different conditions in planktonic and biofilm cultures through 1763 designed probes targeting 105 marker genes associated with motility (flagella and T4P), anaerobic physiology (fermentation and denitrification pathways), stress responses (oxidative and nutrient limitation), biofilm matrix components, quorum-sensing, antibiotic resistance and virulence factors (Dar et al., 2021). The par-seqFISH technique revealed distinct metabolic and virulence-related subpopulations in planktonic growth conditions, as well as highly spatially resolved metabolic heterogeneity in sessile populations. Specifically, they found differences in spatial architecture distinguishing early and mature biofilms, defined by distinct expression patterns of proteases and quorum-sensing regulatory networks (Dar et al., 2021).

The major strength of par-seqFISH is its ability to reveal spatial dynamics by preserving the physical context of bacteria. However, while it offers a powerful method for spatially resolved transcriptomics, it still relies on pre-designed probes targeting specific genes, requiring prior knowledge of the genome of interest to enable probe design. This inherently limits the number of genes that can be queried, restricting its capacity for unbiased discovery.

While par-seqFISH provides spatially resolved transcriptomic information, its multiplexing capacity remains constrained by probe design and imaging cycles. The method is technically demanding and requires extensive probe libraries and advanced microscopy infrastructure. Moreover, because it targets predefined genes, it does not provide full transcriptome coverage and may overlook unexpected regulatory pathways.

### 4.2 Fluorescent reporter systems

Despite recent advances in scRNA-seq techniques, most studies of single-cell gene expression in bacteria still rely on fluorescent protein reporters. When combined with flow cytometry or live-cell time-lapse fluorescence microscopy, these transcriptional and translational reporter systems enable the real-time tracking of gene expression at the single-cell level, revealing phenotypic heterogeneity within populations in space and time. In some cases, fluorescence-activated cell sorting (FACS) can further separate phenotypically distinct subpopulations for downstream analysis.

Recent applications of these reporter systems have provided insights into the bistable expression of *Salmonella enterica* pathogenicity island 1 (SPI-1) and its flagellar network (Flag). Tracking the ON and OFF states of SPI-1 and Flag revealed independent switching, resulting in four distinct subpopulations: SPI-1OFF FlagOFF, SPI-1OFF FlagON, SPI-1ON FlagOFF, and SPI-1ON FlagON. The subsequent sorting and invasion assays showed that none of these subpopulations were highly invasive (Sánchez-Romero and Casadesús, 2021). Similarly, in *P. aeruginosa*, fluorescent reporters revealed heterogeneous expression of T3SS master regulator ExsA. Even in the absence of T3SS-activating signals, a subpopulation of “primed” cells express ExsA, allowing them to rapidly upregulate the transcription of T3SS genes upon stimulation. This phenotypic heterogeneity benefits the population as a whole, as T3SS effectors act as public goods for non-producing cells (Lin et al., 2021).

While fluorescent reporters are powerful and cost-effective tools to dissect single-cell gene expression dynamics and reveal heterogeneity in bacterial populations, each technique used to analyze reporter signals has key limitations. Flow cytometry enables the quantification of gene expression across thousands of cells, offering high-throughput resolution, but only provides a snapshot of cellular states at a single time points. In contrast, fluorescence microscopy can track gene expression dynamics in individual cells over time, but only monitors a small population of bacteria and lacks environmental control. The integration of fluorescent reporter systems with microfluidic platforms has emerged as an effective strategy to overcome these limitations and study bacterial behavior at the single-cell level. Microfluidic platforms confine individual cells within microchambers, microchannels or droplets, enabling precise control over the microenvironment, including oxygen and nutrient gradients, chemical signals, pH levels and temperature, providing high temporal and spatial resolution (Pérez-Rodríguez et al., 2022; Ugolini et al., 2024). This confinement enables continuous, long-term tracking of individual bacterial responses, such as bacterial growth, division, motility and intercellular interactions under controlled and reproducible conditions. Integrated systems provide resolution to dissect complex bacterial behaviors such as antibiotic tolerance (Alnahhas et al., 2025; Moreno-Gámez et al., 2021), biofilm formation (Straub et al., 2020), and quorum-sensing dynamics (Ábrahám et al., 2024) within structured microenvironments.

Fluorescent reporter systems are typically limited to monitoring one or a small number of genes at a time, restricting their capacity to capture genome-wide transcriptional states. Reporter constructs may also perturb native regulatory networks or alter gene expression levels. Furthermore, fluorescence signals often reflect promoter activity rather than absolute transcript abundance, which can complicate quantitative comparisons across cells.

### 4.3 Stable isotope probing at the single-cell level (SC-SIP)

Bacterial heterogeneity can also be explored through single-cell stable isotope probing (SC-SIP), which encompasses techniques such as nano-scale secondary ion mass spectrometry (NanoSIMS) and Raman microspectroscopy. These methods enable spatially resolved tracking of isotope tracers in individual cells, providing detailed insights into metabolic activity and growth heterogeneity within microbial populations (Alcolombri et al., 2022).

Nano-scale secondary ion mass spectrometry employs a primary ion beam, typically composed of cesium or oxygen ions, to sputter the sample surface and release secondary ions that are further collected by an electric field and analyzed by mass spectrometry. NanoSIMS provides high sensitivity and subcellular resolution, allowing precise quantification of isotopic composition in individual cells. However, it is destructive and precludes downstream applications, such as bacterial cultivation or genome sequencing (Alcolombri et al., 2022; Gao et al., 2016; Musat et al., 2012). Leveraging its high spatial resolution, NanoSIMS has revealed phenotypic heterogeneity in sugar metabolism within clonal *E. coli* populations grown in chemostats with ^13^C- or ^2^H-labeled sugars. Individual bacterial cells show variation both in overall sugar assimilation rates and sugar-specific assimilation, resulting in differences in single-cell growth rates (Nikolic et al., 2017). Similarly, in *Klebsiella oxytoca*, NanoSIMS demonstrated that ammonium limitation induces a heterogeneous nitrogen acquisition strategy, with part of the population fixing N_2_ while the remaining one uses NH_4_^+^ (Schreiber et al., 2016).

In contrast, the Raman microspectroscopy is a non-destructive method that couples microscopy with vibrational spectral analysis, producing a biochemical “fingerprint” of each cell. This technique can detect isotopic incorporation, for example, using D_2_O as a universal metabolic tracer. Raman spectroscopy has been applied to differentiate physiological states, such as to distinguish vegetative cells from dormant spores in *Bacillus thuringiensis* (Chen et al., 2006). It has also been used to study *E. coli* persister cells: after treatment with lethal doses of ampicillin, Raman spectra revealed differences in band intensities corresponding to major cellular components and metabolites, indicating that *E. coli* persisters exhibit higher metabolic activities than untreated populations (Wang et al., 2022).

Single-cell stable isotope probing is often used as a complementary technique to gain a more comprehensive understanding of bacterial single-cell physiology. Despite their powerful ability to resolve metabolic heterogeneity, isotope-based approaches provide limited information about the underlying genetic or regulatory mechanisms driving these phenotypes. NanoSIMS is destructive and requires specialized instrumentation with low sample throughput, whereas Raman microspectroscopy typically has lower spatial resolution and sensitivity. Consequently, SC-SIP techniques are often most informative when combined with complementary genomic or transcriptomic approaches.

## 5 Concluding remarks and future perspectives

The notion that microbial populations behave as uniform collections of identical individuals is increasingly recognized as a misconception, and heterogeneity-generating mechanisms are now understood to be highly interconnected. Stochastic gene expression introduces variability, bistable regulatory circuits stabilize distinct phenotypes, and quorum sensing and environmental cues modulate subpopulation dynamics. Persistence mechanisms generate specialized survival states, while history-dependent behavior links past and present responses. Together, these processes form an integrated regulatory framework driving division of labor, bet-hedging, and population-level robustness during infection.

Single-cell technologies are now revealing how these heterogeneous states emerge within infected hosts. For example, bistable expression of SPI-1 virulence genes in *Salmonella* intestinal infections produces invasive and non-invasive subpopulations that cooperate during infection. Similarly, heterogeneous growth rates observed in intracellular *Mycobacterium tuberculosis* highlight the importance of slow-growing or drug-tolerant subpopulations during antibiotic treatment.

These observations demonstrate that bacterial heterogeneity is not merely a laboratory phenomenon but a defining feature of pathogen behavior within host tissues. Such *in vivo* single-cell observations highlight that rare subpopulations, often undetectable in bulk measurements, can critically shape infection dynamics, persistence, and treatment outcomes.

Bacterial heterogeneity can also actively shape host responses. During infection, distinct subpopulations generate heterogeneous immune stimuli within the same niche. For example, in *Salmonella enterica*, SPI-1/T3SS-1 ON cells promote epithelial invasion and inflammation, whereas OFF cells evade immune detection and exploit the inflammatory environment. Similarly, heterogeneous expression of immunogenic factors such as flagellin enables a balance between immune activation and immune evasion across host compartments. In addition, intracellular persister subpopulations can alter macrophage responses, promoting more permissive host states. These observations indicate that phenotypic heterogeneity not only reflects adaptation to the host but also contributes to host–pathogen interaction dynamics.

Although the traditional study of batch cultures to extrapolate the global behavior of a bacterial population is extremely valuable, it is now crucial to understand how heterogeneities among individual cells affect population dynamics during infection. The single-bacterium approaches described here are promising to understand bacterial heterogeneity and have significantly improved throughput and resolution, but some challenges remain regarding the capture of low-abundance transcripts, standardization of data analysis, and the integration of spatial context.

Despite significant advances, the temporal dimension of heterogeneity is critical for understanding pathogenesis. Heterogeneity is dynamic over time, with subpopulations emerging, transitioning, or persisting during infection. Early stages may favor virulence-expressing cells that promote invasion and inflammation, whereas later stages select for slow-growing or persister subpopulations that tolerate immune pressure and antibiotic exposure. These dynamics contribute to persistence and relapse once stress conditions are relieved. Recent studies further support this framework, showing that phenotypic heterogeneity is shaped by social interactions, spatial structure, and host-induced stress responses. Future work will therefore require integrative approaches combining single-cell transcriptomics, spatial imaging, and computational modeling to develop predictive frameworks of virulence heterogeneity and to identify bacterial subpopulations that drive persistence, immune evasion, and treatment failure. These advances could redirect therapeutic approaches toward targeting individual bacteria or subpopulations with a competitive advantage in biofilm formation, host infection or antibiotic resistance.
